# The DEMS-DOSS study: validating a delirium monitoring tool in hospitalised older adults

**DOI:** 10.1093/ageing/afac012

**Published:** 2022-02-22

**Authors:** Amy Montgomery, Jo-Anne Todd, Cindy Jones, June Koroitamana, Laurie Grealish, Anne Wand, Stephen Billett, Andrew Teodorczuk

**Affiliations:** School of Nursing, Faculty of Science, Medicine and Health, University of Wollongong, New South Wales, 2522, Australia; Department of Aged Care, St. George Hospital, Kogarah, New South Wales 2217, Australia; Illawarra Health and Medical Research Institute, University of Wollongong, Wollongong, New South Wales 2522, Australia; Menzies Health Institute Queensland, Griffith University, Queensland 4111, Australia; Faculty of Health Sciences and Medicine, Bond University, Robina, Queensland 4226, Australia; Healthcare Practice and Survivorship, Menzies Health Institute Queensland, Griffith University, Nathan, Queensland 4111, Australia; Department of Aged Care, St. George Hospital, Kogarah, New South Wales 2217, Australia; Menzies Health Institute Queensland, Griffith University, Queensland 4111, Australia; Gold Coast Health, Southport, Queensland 4215, Australia; School of Psychiatry, Faculty of Medicine, University of New South Wales, Australia 2052, Australia; Education and Professional Studies, Griffith University, Brisbane, Queensland 4111, Australia; Metro North Mental Health, The Prince Charles Hospital, Brisbane, Queensland 4032, Australia; Faculty of Medicine, University of Queensland, Brisbane, Queensland 4072, Australia; School of Medicine and Dentistry, Griffith University, Queensland 4111, Australia

**Keywords:** delirium, screening, education, monitoring, detection, older people

## Abstract

**Objective:**

to evaluate the sensitivity, specificity and test–retest reliability of the Delirium Early Monitoring System-Delirium Observation Screening Scale (DEMS-DOSS).

**Design:**

prospective diagnostic accuracy study of a convenience sample of admitted older adults with DEMS-DOSS and reference standard assessments.

**Setting:**

60-bed aged care precinct at a metropolitan hospital in Sydney, Australia.

**Participants:**

156 patients (aged ≥65 years old) were recruited to participate between April 2018 and March 2020. One hundred participants were included in the analysis.

**Measurements:**

Participants were scored on the DEMS-DOSS. Trained senior aged care nurses conducted a standardised clinical interview based on the Diagnostic and Statistical Manual of Mental Disorder (DSM)-IV delirium criteria, within two hours of DEMS-DOSS completion. The senior aged care nurse undertaking the DSM-IV interview was blinded to the results of the DEMS-DOSS.

**Results:**

Participants’ mean age was 84 (SD ±7.3) years and 39% (*n* = 39) had a documented diagnosis of dementia. Delirium was detected in 38% (*n* = 38) according to the reference standard. The DEMS-DOSS had a sensitivity of 76.3% and a specificity of 75.8% for delirium. The area under the receiver operating characteristics curve for delirium was 0.76. The test–retest reliability of the DEMS-DOSS was found to be high (*r* = 0.915).

**Conclusion:**

DEMS-DOSS is a sensitive and specific tool to assist with monitoring new onset and established delirium in hospitalised older adults. Further studies are required to evaluate the impact of the monitoring tool on health outcomes.

## Key Points

- Delirium monitoring aims to embed delirium as a vital sign for ongoing assessment to improve detection of delirium.
- Delirium monitoring tools serve as practice-based mediators of work-based learning to upskill ward staff.
- There is a lack of validated delirium monitoring tools, this paper validates the Delirium Early Monitoring System-Delirium Observation Screening Scale (DEMS-DOSS) as a monitoring tool.

## Introduction

Delirium has a high prevalence in older hospitalised adults and is associated with negative healthcare and economic outcomes [1, 2]. Delirium continues to be under-recognised and missed in up to two-thirds of cases, therefore, opportunities to improve the care of hospitalised older adults are also missed [3, 4]. Over the past two decades, the development and introduction of practice guidelines and validated screening tools such as 4AT [5, 6], Nu-DESC [7] and Confusion Assessment Method (CAM) [8] have highlighted the importance of preventing, recognising and treating delirium in clinical settings.

More recently, there is the suggestion that more is needed to improve patient care beyond the initial delirium screening, and delirium monitoring may fill this gap [9]. Delirium monitoring is defined by Marra and colleagues [10] as the ‘initial detection and ongoing evaluation of a patient’s delirium status during a hospital admission by using predefined criteria’. As such it entails both delirium screening and also ongoing assessment of progress and recovery. Monitoring embeds delirium as a vital sign for ongoing assessment and not only improves detection but importantly also understanding of delirium and communication between patients, families and clinicians [10]. Further, a monitoring tool may allow staff to quantify subtle changes in cognition to prompt further assessment for the new onset of delirium. However, there is ongoing debate on the utility of delirium monitoring and challenges in their application in clinical settings [10]. As such in practice, delirium monitoring is rarely undertaken with a lack of validated tools. To date, one specific delirium monitoring tool, Recognising Delirium As part of your Routine, has been validated [11].

The Delirium Early Monitoring System (DEMS) was developed as a monitoring tool to promote ongoing delirium detection and assessment [12]. Crucially, DEMS has a contemporary educational theoretical underpinning and has been designed specifically to facilitate learning through practice [13]. Similar to the Modified Early Warning Scale, it links the scores on monitoring scales directly to actions [14]. This is particularly important as evidence suggests that simple teaching about delirium alone fails to lead to practice change and there is a need for tools such as DEMS to support ongoing learning by initiating actions and, thereby, informing practice changes [13]. Actions are determined according to resources within healthcare setting but may include reporting the finding to the shift coordinator for a medical review within four hours if Delirium is detected [12].

The DEMS was piloted in the North East of England using a modified screening tool [12], Delirium Observation Screening Scale (DOSS) [15, 16]. DOSS was selected because it is an observational delirium scale rather than a subject-based cognitive assessment that may not lend itself to repeated application in a clinical setting. The DEMS-DOSS has been well received and acceptable in practice and noted to facilitate initiation of action triggers as needed [12]. However, the validity and reliability of the DEMS-DOSS as a tool for monitoring delirium has yet to be established. This is an important step that is necessary before further evaluating the impact of DEMS-DOSS on work-based learning, ward culture and healthcare outcomes.

### Aims

The objectives of this study were to evaluate the sensitivity and specificity of DEMS-DOSS as a delirium monitoring tool as well as its test–retest reliability.

### Methods

Prospective diagnostic accuracy study to validate DEMS-DOSS as a delirium monitoring tool in patients aged ≥65 years admitted to aged care wards at a metropolitan hospital in Sydney, Australia, from April 2018 to March 2020. Patients were excluded if they had severe hearing impairment, unable to verbally communicate and/or unable to provide consent or without a recognised substitute decision-maker.

**Figure 1:**
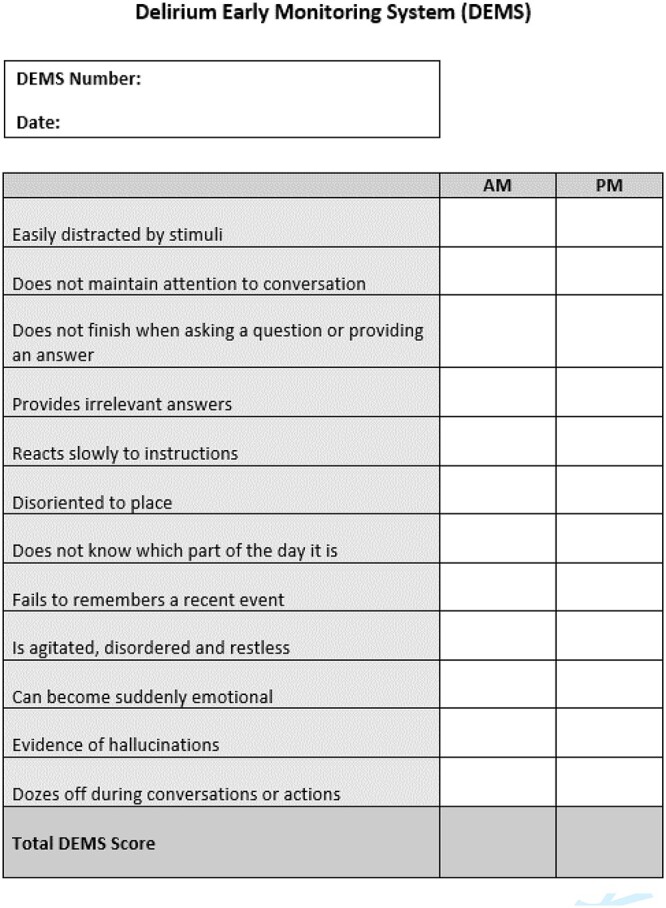
DEMS-DOSS scoring sheet.

### Participants’ recruitment and sample size

Within 72 hours of hospital admission, patients were screened for eligibility by senior aged care nurses Supplementary Appendix 1. Convenience sampling was used. Informed consent to participate was provided and data were collected for each participant over a 24-hour period. Demographic information including gender, primary language, country of origin, number of comorbidities and documented evidence of dementia or other neurodegenerative disease was obtained from the medical records. The target sample size was 120 (i.e. minimum power of 90% to detect a respective sensitivity and specificity values of 0.8–0.9 and 0.5–0.9 of a screening test at *α* = 0.05) [17].

## Instruments

### DEMS-DOSS

A modified version of the 13 item DEMS-DOSS, consisting of 12 items (Figure 1) was completed. Each item was rated as 0 (not observed) or 1 (observed) and a score of >3 on the DEMS-DOSS indicated the presence of possible delirium requiring further investigation. Nursing staff were trained in the use of DEMS-DOSS. Training consisted of a seven-minute video, crib sheets and bedside training with the SACNs.

### Diagnostic and Statistical Manual of Mental Disorders criteria for delirium

The reference standard assessment was the Diagnostic and Statistical Manual of Mental Disorder (DSM-IV) criteria for delirium [18]. This assessment incorporated a clinical interview which focused on alertness, orientation and attention. As there is a variation of opinion among delirium experts as to how the cut-offs of the DSM-IV criteria should be applied [19], the research team determined that a participant positive for two or more criteria would be rated to have likely delirium. The senior aged care nurses with expertise in delirium recognition and diagnosis completed the DSM-IV criteria. The senior aged care nurses were trained and supervised by an aged care nurse practitioner. Prior to the commencement of the study, the senior aged care nurses independently assessed a subgroup of patients (*n* = 3) to check inter-rater reliability using the DSM-IV criteria. A level of 100% agreement was reported for each item.

### Procedures

Participants were scored once on DEMS-DOSS between 8 and 10 am, the morning after recruitment to the study (Time A) (Figure 1). To determine test–retest reliability, at least 25% of participants were rated on DEMS-DOSS by a second nurse within a two-hour timeframe (Time B). A trained senior aged care nurse, blinded to the DEMS-DOSS results but not blinded to clinical information, conducted a standardised clinical interview based on the DSM-IV delirium criteria, within two hours of DEMS-DOSS (Time A) completion Supplementary Appendix 1.

### Statistical analysis

Statistical analysis was conducted using IBM SPSS Version 26 [20]. Descriptive statistics provided an overview of participants’ demographics. Pearson’s *r* and Cronbach’s *α* were calculated to evaluate the test–retest and internal reliability of the DEMS-DOSS. Receiver operating characteristic (ROC) was computed to ascertain the diagnostic accuracy of DEMS-DOSS to yield sensitivity, specificity and area under the ROC curve. Data from participants who completed both DEMS-DOSS and DSM-IV assessments were included for analysis. Approval was received from the Human Research Ethics Committees of Griffith University and South Eastern Sydney Local Health District (17/218).

## Results

In total, 156 older hospitalised adults consented to participate in the study; however, only 100 participants were included in the analysis. The reasons participant data were excluded from the study were; discharged before ‘DEM-DOSS competed’ (*n* = 18), ‘DEMS-DOSS’ not completed within 48 hours (*n* = 24), DSM-IV not completed within correct timeframe (*n* = 1), incomplete or missing data (*n* = 2) and others reasons (transferred to another ward or decreased) (*n* = 11) (Supplementary Appendix S2). Supplementary Appendix S3 shows the demographic characteristics of participants. The mean age was 84 years (SD ± 7.3), 39% (*n* = 39) had an existing dementia diagnosis and 49.0% (*n* = 49) had seven or more comorbidities. The study included culturally and linguistically diverse older adults, with 55% (*n* = 55) reporting English as their first language and the remaining as, Greek (20%), Italian (10%) and others (16%). Around 38% were evaluated as being positive for delirium based on the DSM-IV.

**Figure 2:**
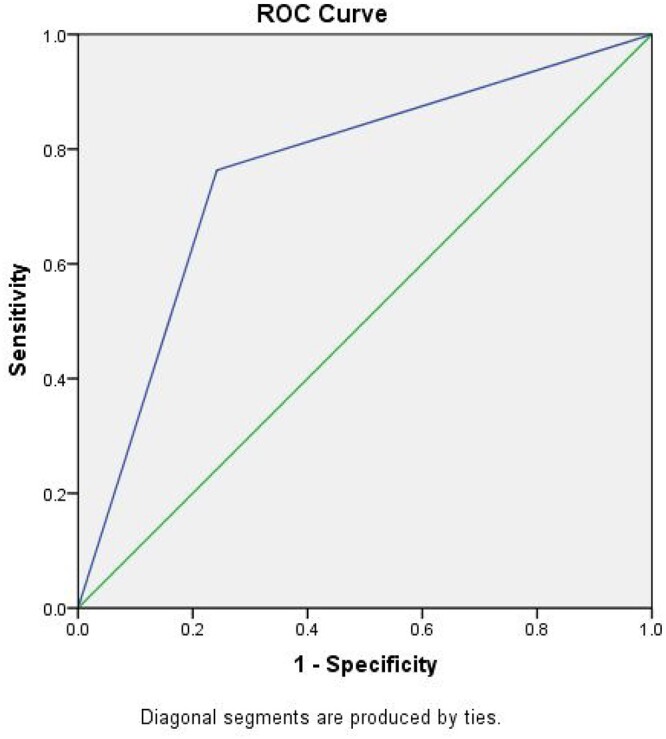
Receiver operating characteristic (ROC) curve for the DEMS-DOSS versus reference standard the DSM-IV delirium criteria.

The sensitivity and specificity of DEMS-DOSS for delirium were 76.3 and 75.8%, respectively. The ROC is depicted in Figure 2. The area under the curve was 0.76 (95% CI: 0.66–0.86) which is deemed as fair/acceptable [21]. Cronbach’s *α* for internal consistency was good; 0.84. Overall, the test–retest reliability of DEM-DOSS was found to be high (*r* = 0.92).

## Discussion

Previously, we have demonstrated that DEMS-DOSS as a monitoring tool is easy to use in practice [12]. We now report evidence that DEMS-DOSS is a valid and reliable tool that can, therefore, be considered for introduction into practice for the monitoring of delirium in hospitalised older adults. This is an important finding as, currently, there is a lack of validated monitoring tools and a greater need for delirium monitoring to guide effective delirium practice beyond screening alone. The added value of monitoring tools is that they have the ability to become more embedded within the practice and therefore hold the promise of greater yield in terms of driving work-based learning and practice change [13].

The specificity of 76% for DEMS-DOSS is less than that reported for diagnostic tools such as CAM and 4AT [5, 6, 8]. However, it is important to note that delirium monitoring tools serve a different purpose than screening tools. Their value is derived from ease of use and educational impact to sustain changes in clinical practice. In this regard, monitoring tools serve as practice-based mediators of work-based learning to upskill ward staff and develop a new discourse, in clinical spaces [22].

This study has several strengths, including being pragmatic, real world and with limited exclusion criteria. The senior aged care nurses conducting the DSM-IV were experts in delirium recognition and were blinded to DEMS-DOSS scores. The delirium rate of 37.3% aligns with the estimated prevalence of delirium among older inpatients [23]. We acknowledge the following limitations of this study. While we aimed to ascertain dementia status from the participants’ medical history, this was likely an underestimate as dementia—like delirium—is underdiagnosed [24]. There was a smaller than expected sample size. This sampling reflects the challenges of conducting research in an acute care setting, as 18.5% of recruited participants were discharged, transferred to another ward or died prior to data collection being completed (Supplementary Appendix S2). However, delirium research is known to be challenging and the recruitment difficulties encountered are not unexpected [25].

This study is an important step leading to the implementation into the practice of a tool that could change delirium monitoring practices. Future research should investigate the impact of DEMS-DOSS on healthcare outcomes, ward culture and developing effective delirium practice within hospital settings.

## Supplementary Material

aa-21-1508-File002_afac012

## References

[1] Witlox J, Eurelings L, de Jonghe J, Kalisvaart K, Eikelenboom P, van Gool W. Delirium in elderly patients and the risk of postdischarge mortality, institutionalization, and dementia: a meta-analysis. JAMA 2012; 304: 443–51. 10.1001/jama.2010.1013 20664045

[2] Pezzullo L, Streatfeild J, Hickson J, Teodorczuk A, Agar M, Caplan G. Economic impact of delirium in Australia: a cost of illness study. BMJ Open 2019; 9: e027514. 10.1136/bmjopen-2018-027514 PMC6756419 31530588

[3] Casey P, Cross W, Mart MW, Baldwin C, Riddell K, Dārziņš P. Hospital discharge data under-reports delirium occurrence: results from a point prevalence survey of delirium in a major Australian health service. Intern Med J 2019; 49: 338–44. 30091294 10.1111/imj.14066

[4] Malik A, Harlan T, Cobb J. Stop. Think. Delirium! A quality improvement initiative to explore utilising a validated cognitive assessment tool in the acute inpatient medical setting to detect delirium and prompt early intervention. J Clin Nurs 2016; 25: 3400–8. 27105295 10.1111/jocn.13166

[5] Shenkin SD, Fox C, Godfrey M et al. Delirium detection in older acute medical inpatients: a multicentre prospective comparative diagnostic test accuracy study of the 4AT and the confusion assessment method. BMC Med 2019; 17: 138–51. 31337404 10.1186/s12916-019-1367-9 PMC6651960

[6] Tieges Z, MacLullich AMJ, Anand A et al. Diagnostic accuracy of the 4AT for delirium detection in older adults: systematic review and meta-analysis. Age Ageing 2021; 50: 733–42. 33951145 10.1093/ageing/afaa224 PMC8099016

[7] Gaudreau J-D, Gagnon P, Harel F, Roy M-A. Impact on delirium detection of using a sensitive instrument integrated into clinical practice. Gen Hosp Psychiatry 2005; 27: 194–9. 15882766 10.1016/j.genhosppsych.2005.01.002

[8] Wei L, Fearing M, Sternberg E, Inouye S. The confusion assessment method: a systematic review of current usage. J Am Geriatr Soc 2008; 56: 823–30. 18384586 10.1111/j.1532-5415.2008.01674.x PMC2585541

[9] Wilson JE, Mart MF, Cunningham C et al. Delirium. Nat Rev Dis Prim 2020; 6: 90. 33184265 10.1038/s41572-020-00223-4 PMC9012267

[10] Marra A, Kotfis K, Hosie A et al. Delirium monitoring: Yes or no? That is the question. Am J Crit Care 2019; 28: 127–35. 30824517 10.4037/ajcc2019874

[11] Voyer P, Champoux N, Desrosiers J et al. Recognizing acute delirium as part of your routine [RADAR]: a validation study. BMC Nurs 2015; 14: 19–131. 25844067 10.1186/s12912-015-0070-1 PMC4384313

[12] Rippon D, Milisen K, Detroyer E et al. Evaluation of the delirium early monitoring system (DEMS). Int Psychogeriatrics 2016; 28: 1879–87. 10.1017/S1041610216000983 27443322

[13] Teodorczuk A, Billett S. Mediating workplace situational pressures: the role of artefacts in promoting effective interporfessional work and learning. Focus Heal Prof Educ A Multi-Disciplinary J 2016; 18: 80–91.

[14] Subbe C, Kruger M, Rutherford P, Rutherford L. Validation of a modified early warning score in medical admissions. QJM An Int J Med 2001; 94: 521–6. 10.1093/qjmed/94.10.521 11588210

[15] Schuurmans MJ, Shortridge-Bagget LM, Duursma SA. The Delirium Observation Screening Scale: a screening instrument for delirium. Res Theory Nurs Pract 2003; 17: 31–50. 12751884 10.1891/rtnp.17.1.31.53169

[16] Scheffer AC, Van Munster BC, Schuurmans MJ, De Rooij SE. Assessing severity of delirium by the Delirium Observation Screening Scale. Int J Geriatr Psychiatry 2011; 26: 284–91. 20665557 10.1002/gps.2526

[17] Bujang MA, Adnan TH. Requirements for minimum sample size for sensitivity and specificity analysis. J Clin Diagnostic Res 2016; 10: YE01–06. 10.7860/JCDR/2016/18129.8744 PMC5121784 27891446

[18] American Psychiatric Association . Diagnostic and Statistical Manual of Mental Disorders. 4th edition. Washington, DC: Author, 2020.

[19] Meagher DJ, Morandi A, Inouye SK et al. Concordance between DSM-IV and DSM-5 criteria for delirium diagnosis in a pooled database of 768 prospectively evaluated patients using the delirium rating scale-revised-98. BMC Med 2014; 12: 165–73. 25266390 10.1186/s12916-014-0164-8 PMC4207319

[20] IBM Corp . IBM SPSS Statistics for Windows, Verison 26.0. Armonk, NY: IBM Corp, 2019.

[21] Hosmer D, Lemeshow S, Sturdivant R. Applied Logistic Regression. 3rd edition. Hoboken, NJ: John Wiley & Sons, Ltd, 2013.

[22] Lee SY, Fisher J, Wand APF et al. Developing delirium best practice: a systematic review of education interventions for healthcare professionals working in inpatient settings. Eur Geriatr Med 2020; 11: 1–32. 10.1007/s41999-019-00278-x 32297244

[23] Koirala B, Hansen B, Hoise A et al. Delirium point prevalence studies in inpatient settings: a systematic review and meta-analysis. J Clin Nurs 2020; 29: 2083–92. 32065410 10.1111/jocn.15219

[24] Lang L, Clifford A, Wei L et al. Prevalence and determinants of undetected dementia in the community: a systematic literature review and a meta-analysis. BMJ Open 2017; 7: e011146. 10.1136/bmjopen-2016-011146 PMC5293981 28159845

[25] Harwood R, Teale E. Where next for delirium research? Int J Geriatr Psychiatry 2017; 33: 1512–20. 28271556 10.1002/gps.4696

