# Supplementary material for: The DEMS-DOSS study: validating a delirium monitoring tool in hospitalised older adults
Source: Age Ageing. 2022 Feb 22;51(2):afac012. doi: 10.1093/ageing/afac012 (PMC9171726; doi:10.1093/ageing/afac012)
Supplement: aa-21-1508-File002_afac012 [file aa-21-1508-file002_afac012.docx]

**The DEMS-DOSS study: Validating a delirium monitoring tool in hospitalised older adults.**

**Appendix:**

**Appendix A: Participant Timeline**

**Appendix B: Participant Recruitment**

**Appendix C: Demographic characteristics of participants (n=100)**

**Appendix A: Participant Timeline**


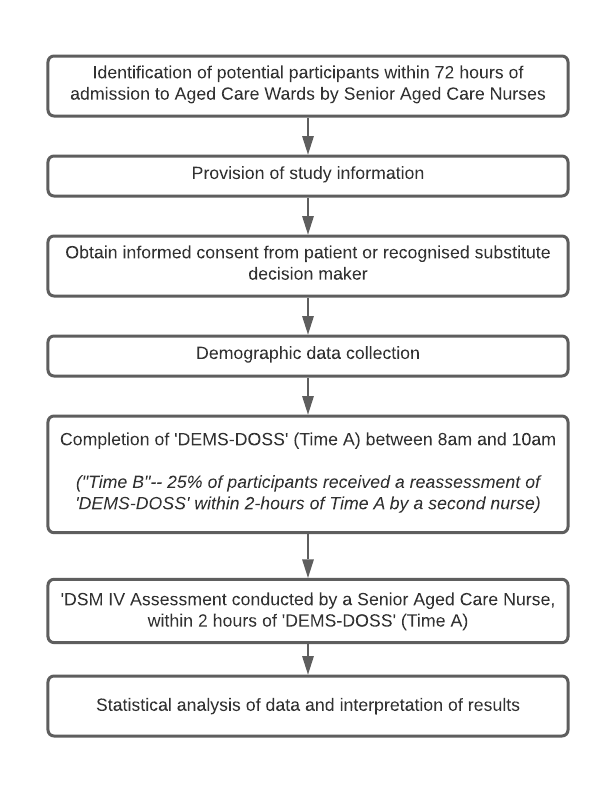


**Appendix B: Participant Recruitment**


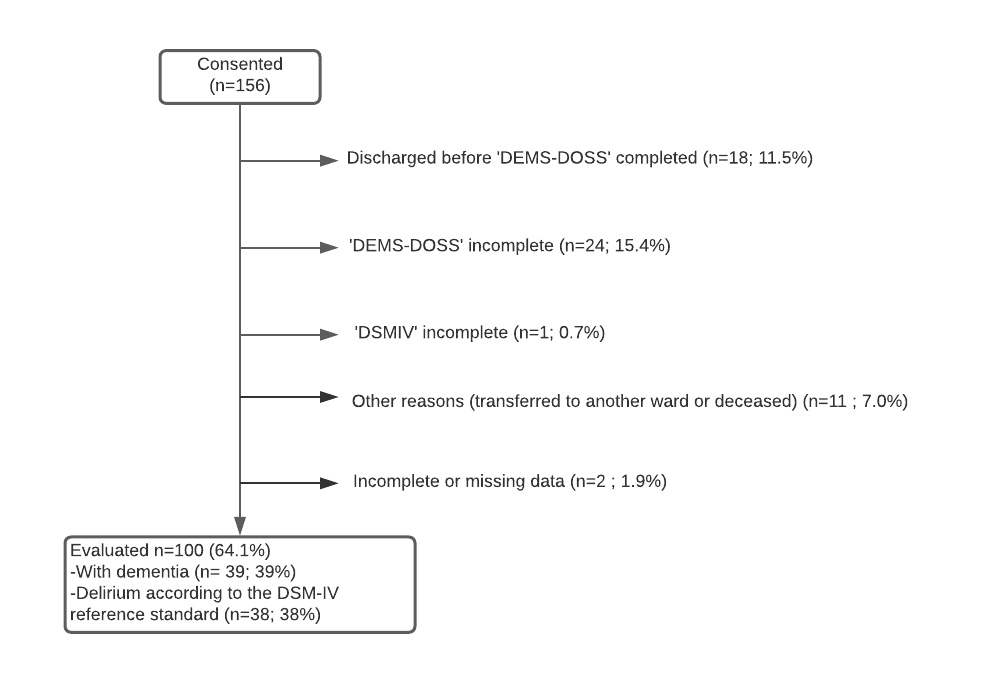


**Appendix C: Demographic characteristics of participants (n=100)**

|  | N (%) | M (SD) |
| --- | --- | --- |
| Age (years) |  | 84 (7.3) |
| Sex (male) | 54 (54) |  |
| Dementia Diagnosis | 39 (39) |  |
| Country of Birth (Australia) | 50 (50) |  |
| Country of Birth other than Australia | 52 (52) |  |
| 7 or more comorbidities | 50 (50) |  |
| DSM-IV Delirium Probable | 38 (38) |  |

**M (SD): Mean (Standard Deviation)**
